# *Notes from the Field:* Chikungunya Outbreak — Paraguay, 2022–2023

**DOI:** 10.15585/mmwr.mm7223a5

**Published:** 2023-06-09

**Authors:** Martha Torales, Amy Beeson, Lorena Grau, Miguel Galeano, Andrea Ojeda, Bettiana Martinez, Nancy León, Agueda Cabello, Fátima Rojas, Viviana de Egea, Rosa Galeano, Sandra Ocampos, Cynthia Vazquez, Romeo Montoya, Susan Hills, Guillermo Sequera

**Affiliations:** ^1^General Directorate of Health Surveillance, Ministry of Public Health and Social Welfare, Asunción, Paraguay; ^2^Division of Vector-Borne Diseases, National Center for Emerging and Zoonotic Infectious Diseases, CDC; ^3^Epidemic Intelligence Service, CDC; ^4^Central Public Health Laboratory, Ministry of Public Health and Social Welfare, Asunción, Paraguay; ^5^Pan American Health Organization, Asunción, Paraguay; ^6^Faculty of Medical Sciences, National University of Asunción, Asunción, Paraguay.

Local transmission of chikungunya virus (CHIKV) was first reported in the Americas during December 2013, followed by widespread regional transmission (*1*). CHIKV is transmitted primarily by *Aedes aegypti* and *Aedes albopictus* mosquitoes. Most infected persons (72%–97%) experience symptomatic illness, typically including fever and often severe polyarthralgia (which can persist for months or years) (*2*). Rare complications include neurologic, cardiac, or renal disease (*2*).

Paraguay reported its first autochthonous chikungunya case during 2015 (*3*). The subsequent outbreak, concentrated in the capital city of Asunción and the neighboring Central Department, resulted in 5,221 cases during 2015–2016.* A second outbreak (1,239 cases) occurred during 2018 in the north-central Amambay Department. Beginning the first week of October 2022, an increase in reported cases was again noted; this report provides preliminary information on this outbreak as of March 11, 2023.

During October 1, 2022–March 11, 2023, a total of 81,037 suspected, probable, or confirmed^†^ chikungunya cases was recorded by the Paraguayan Ministry of Health^§^; among these, 75,911 (94%) occurred during 2023. Most cases occurred in Central Department (49,070; 61%) and Asunción (16,094; 20%). Cumulative national incidence was 1,073 cases per 100,000 population (3,088 per 100,000 population in Asunción).^¶^ Weekly case counts in Asunción and Central Department declined slightly after epidemiologic week 6, but an increasing number and proportion of cases were subsequently reported from outlying regions, including along borders with Brazil and Argentina.

Among 47,116 probable or confirmed cases,** 27,147 (58%) were in females, and the median age was 36 years (range = 0 days–103 years); 4,604 (10%) hospitalizations and 52 (<1%) deaths attributable to CHIKV infection were reported.^††^ Among 208 (0.4%) cases in infants aged ≤29 days (neonates), 140 hospitalizations and eight deaths were reported,^§§^ accounting for the highest case fatality rate (3.8%) among all age groups (Figure). Among fatal neonatal cases, the timing of symptom onset suggested intrapartum transmission in 75% and mosquitoborne transmission in 25%.^¶¶^ Among adults aged ≥60 years, 10,617 cases and 1,878 hospitalizations (41% of all hospitalizations) were reported. Within this group, 32 deaths occurred***; 23 (72%) and 13 (41%) decedents had documented cardiovascular disease and diabetes, respectively, and 20 (63%) had two or more comorbidities. The highest case fatality rate among adults aged ≥60 years occurred among those aged ≥80 years (0.6%; 11 of 1,719 cases).

**FIGURE:**
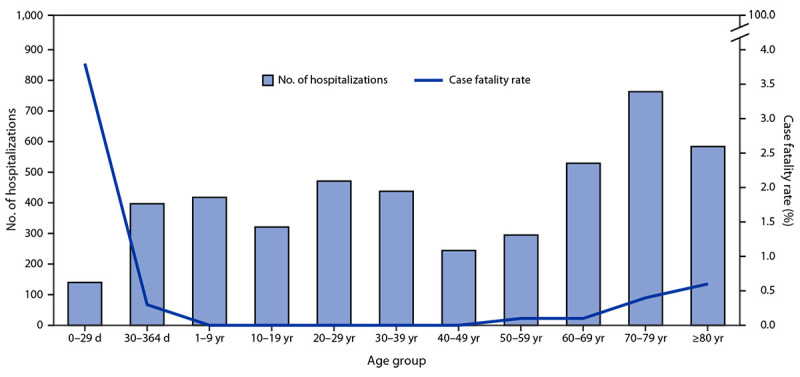
Number of hospitalizations (N = 4,604) and case fatality rate* among probable and confirmed chikungunya cases, by age group — Paraguay, October 1, 2022–March 11, 2023 * Deaths per 100 cases.

CHIKV can cause explosive outbreaks and substantial morbidity in all age groups. Groups at risk for severe disease and death include older adults, persons with comorbidities, and infants. Prevention messages should focus on avoiding mosquito bites by wearing long-sleeved shirts and pants, using insect repellents, screening windows and doors, and covering cribs, strollers, and beds with netting. Mosquito breeding sites around homes should be eliminated by emptying, scrubbing, or covering water-holding containers. In addition, integrated vector surveillance and control measures are essential at the community level. Persons with suspected infection should prevent mosquito bites to reduce spread to others. These measures will also reduce the risk for infection from dengue and Zika viruses, which often co-circulate; dengue virus is currently co-circulating in Paraguay.

No antiviral treatment for chikungunya exists; however, timely dissemination of diagnosis and management guidance is crucial.^†††^ Newborns with possible intrapartum exposure should be monitored in a hospital during the first week of life, because deterioration can occur rapidly (*4*). Infection prevention measures should be implemented in hospitals to limit spread to staff members and patients, including providing bed nets and repellent for inpatients with chikungunya and eliminating mosquito breeding sites from hospital grounds.

Humans are the primary reservoir during epidemics and can transport the virus to new areas; cases in travelers returning from Paraguay have been reported in several countries^§§§^ (*5*). If an infected person is bitten by a mosquito vector at their destination, a risk for local transmission exists. During 2022–2023, in the Americas, increases in chikungunya cases and spread outside historical transmission areas (e.g., Uruguay and parts of Argentina) have occurred.^¶¶¶^ Strengthened surveillance and preparedness are crucial (*5*). Although no vaccine is currently licensed, several are in the late stages of development and could have a role in reducing cases and deaths in future outbreaks (*2*).

## Footnotes

*
https://www.mspbs.gov.py/dependencias/portal/adjunto/0a6e46-SE82023Alerta.pdf

† On the basis of the Paraguayan Ministry of Public Health and Social Welfare’s case definition, a suspected case is illness in a person with abrupt onset of a temperature ≥99.5°F (≥37.5°C) and incapacitating arthralgia or arthritis not explained by another medical condition, or in a neonate with fever, irritability, or cutaneous eruption. A probable case is a clinically compatible case with an epidemiologic link to a confirmed case or a positive CHIKV immunoglobulin (Ig) M test result. A confirmed case is any case with evidence of CHIKV infection determined by reverse transcription–polymerase chain reaction (RT-PCR) testing or virus isolation.

§ The number of suspected cases reported here excludes cases that were discarded on the basis of a negative RT-PCR test result <7 days, or a negative IgM test result ≥7 days, after symptom onset.

¶ Incidence calculated using 2023 population projections from the Paraguayan National Census. https://www.ine.gov.py/microdatos/index.php?cant=99&tema=TODOS

** Probable and confirmed cases were used to describe demographic and clinical findings because data were more complete.

†† Pediatric and adult mortality committees met weekly to review comprehensive clinical information on deaths of patients with probable or confirmed chikungunya and classify the cause of death as attributable to 1) CHIKV infection, or 2) another cause in a patient with concurrent CHIKV infection.

§§ An additional eight deaths of neonates with probable or confirmed chikungunya were determined not attributable to chikungunya, and eight deaths among suspected, probable, or confirmed cases were pending a review of cause of death or laboratory results to confirm infection.

¶¶ Among eight neonates who died, symptom onset after birth was <7 days for six cases and >14 days for two cases.

*** An additional 136 deaths among persons aged ≥60 years with probable or confirmed chikungunya were determined not attributable to chikungunya, and 163 deaths among suspected, probable, or confirmed cases were pending a review of cause of death or laboratory results to confirm infection.

†††
https://www.paho.org/en/documents/preparedness-and-response-chikungunya-virus-introduction-americas

§§§
https://www.argentina.gob.ar/salud

¶¶¶ https://www.paho.org/en/documents/epidemiological-alert-increase-cases-and-deaths-chikungunya-region-americas
